# Metallurgical Process Simulation and Optimization

**DOI:** 10.3390/ma15238421

**Published:** 2022-11-26

**Authors:** Jiangshan Zhang, Yuhong Liu, Qing Liu

**Affiliations:** State Key Laboratory of Advanced Metallurgy, University of Science and Technology Beijing, Beijing 100083, China

Metallurgy involves the art and science of extracting metals from their ores and modifying them for use. Over thousands of years of development, many interdisciplinary technologies have been introduced into this large-scale, traditional industry. In modern metallurgical practices, modeling and simulation have been widely used to provide solutions for design, control, optimization, and visualization, and they are increasingly significant in the progress of digital transformation and intelligent metallurgy. This Special Issue (SI), “Metallurgical Process Simulation and Optimization”, presents recent advances in the modeling and optimization of metallurgical process, and includes nearly twenty articles covering various aspects of the topic. A second volume of this successful SI is being organized in which researchers from both academia and industry are invited to publish their new work. The purpose of the current editorial is to briefly summarize the publications included in this SI.

Advanced modeling methods have been widely used to simulate the fluid flow, heat transfer, and mass transfer behavior of metallurgical vessels, and are usually coupled with species, multiphase, and electromagnetic fields to optimize their design/operation. The effect of interfacial tension on alumina inclusion motion behavior was studied in a continuous casting mold using a commercial CFD software package, as reported by Siddiqui et al. [1]. It was found that inclusions were vulnerable to engulfment by the solidification front under a high-surface-tension gradient. Tie et al. [2] shed light on the fluid flow inside an asymmetric multi-strand tundish using both numerical and physical simulation, and the flow field was optimized to improve the overall quality of bloom castings and con-rod products. In Zhao et al.’s [3] numerical study of mesoscopic fluid-particle flow, a bifurcated pool-type SEN under steady operating conditions was employed using the lattice Boltzmann method (LBM) coupled with the large eddy simulation (LES) model with validation. Liu et al. [4] investigated the influence of M-EMS on fluid flow and initial solidification in slab continuous casting; a model coupled with an electromagnetic field was developed to simulate the transient turbulence flow and initial solidification, with special consideration of the effects of different electromagnetic stirring (EMS) currents and casting speeds. The function of an Electric Arc Furnace is essentially to melt metals, and Moskal et al. [5] optimized the melting process using statistical-thermodynamic modeling based on, among other things, multiple linear regression (MLR). In the work of Duan et al. [6], the gas–solid heat transfer and decomposition processes of limestone calcined with blast furnace gas were studied in a parallel-flow regenerative lime kiln; a Porous Medium Model (PMM) and a Shrinking Core Model (SCM) were used to examine the feasibility of calcining limestone with low-calorific fuel gas. The software ANSYS was used in Deng et al.’s [7] study to simulate the temperature field and stress–strain distribution on the working layer of a four-strand tundish under three preheating stages through the indirect coupling method.

State-of-the-art characterization techniques and experimental methods enable more detailed and accurate insights into the metallurgical process, and provide enriched information for modeling and process optimization. Li et al. [8] examined the nucleation process of zirconium oxide inclusions in steel using classical nucleation theory and first principles, accompanied by SEM, TEM, and XRD characterization methods. An optimized nucleation pathway of ZrO2 was achieved at a high temperature. Molecular dynamics simulations (MD) were combined with experiments in Du et al.’s work [9], which revealed the existence of the form and functional mechanism of CaF2 in phosphosilicate systems. The hot deformation characteristics of Ti alloys were analyzed using the strain hardening exponent, strain rate sensitivity, a processing map, and microstructure observation in Piao et al.’s study [10], and an optimum processing temperature and strain rate were proposed. Wang et al. [11] developed an in situ observation method to characterize the solidification of duplex steel during solidification, and phase-field simulation was also carried out to study its solidification and heat transfer behavior. The influence of the mineralogical structure of mold flux film on heat transfer was studied during continuous casting of peritectic steel in the work of Liu et al. [12], which covers the layered structure, crystallization ratio, mineralogical species, and morphology features of flux films. Optimized mineral phase structures of flux film were put forward to eliminate longitudinal cracks. An electro-gas welding experiment on thickness E36 steel plates was conducted in Fu et al.’s study [13]. A semi-ellipsoid heat source model was developed using linear, sinusoidal, or oscillate-stop paths. Different heating paths were recommended for corresponding thicknesses of steel plate. Malinowski et al. [14] investigated the beneficial effect of rare-earth metal oxides on the wear resistance of surface layers applied to castings, which are intended for structural elements of machinery and equipment in mining and recycling. Exploratory data analysis was carried out for the evaluation of tribological properties, and improvements were made to the modified surface wear resistance. The effects of another rare-earth metal, Cerium (Ce), were studied on the casting slab quality, microstructure and inclusions of cryogenic vessel steel in Wu et al.’s work [15]. Moreover, Zaba et al. [16] used full-field image correlation and infrared thermography techniques to study the effects of strain rate, specimen orientation, and plastic strain on the value and distribution of temperature in dog-bone stainless-steel specimen deformation in uniaxial tensile tests. In addition, Liu et al. [17] used positron annihilation lifetime spectroscopy (PALS), high-resolution transmission electron microscopy (HRTEM), and positron annihilation Coincidence Doppler broadening (CDB) techniques synergistically to study the microstructural evolution of alloys in their early aging stages and at low temperatures. Moreover, an optimized measurement temperature was proposed.

In addition, thermodynamic analysis of H_2_ behavior was conducted in an iron-making blast furnace at different stages of the process of integrating top-gas recycling and CO_2_ electrolysis for H_2_-rich gas injection, as discussed in Hu et al.’s study [18]; this is of fundamental importance for better performance in H_2_ metallurgy.
